# BIRTHPLACE AND COGNITION AMONG US OLDER ADULTS: EVIDENCE FROM THE HARMONIZED COGNITIVE ASSESSMENT PROTOCOL (HCAP)

**DOI:** 10.1093/geroni/igad104.0612

**Published:** 2023-12-21

**Authors:** Zhuoer Lin, Xi Chen

**Affiliations:** Yale University, New Haven, Connecticut, United States; Yale University, New Haven, Connecticut, United States

## Abstract

Growing evidence shows that place of birth and related circumstances may impose lasting and multiplicative impacts on a host of late-life outcomes; yet how it affects various domains of cognitive function in late-life is less understood. In this paper, we exploit the comprehensive cognitive assessments in the Health and Retirement Study (HRS) Harmonized Cognitive Assessment Protocol (HCAP) and HRS geographic data at birth to determine to what extent birth location measured at the state level is associated with cognitive status in later life for a representative sample of Americans over age 50 (N=3,324). We found that state of birth significantly influenced various cognitive domains later in life (including episodic memory, executive function, attention, visuospatial function, language fluency and orientation); and the regional rankings tended to be highly consistent across the cognitive assessments in each domain. Regression analysis demonstrated that the geographic disparities in cognition across birth states could be partly explained by the late-life differences in socioeconomic statuses. Nevertheless, Shapley decompositions demonstrated that, after accounting for mid-life to late-life factors, state of birth still contributed 20%-30% of the explained variations in cognitive outcomes. Particularly, foreign-born individuals and those born in the southern states tended to score the lowest on average on each of the cognitive tests/domains and can account for most of the explained differences in cognition. These findings suggest that place of birth may have lasting impacts on disparities in cognition later in life. Public policy should play pivotal roles in leveling the playing field in early life.

